# Protection From Demyelination by the Novel Adenosine Dual A_2A_ /A_2B_ Receptor Antagonist P626 in EAE and Cultured Oligodendrocyte Precursor Cells

**DOI:** 10.1111/jcmm.70952

**Published:** 2025-11-17

**Authors:** M. Morozzi, F. Cherchi, C. Sasia, L. Frulloni, G. Videtta, M. Venturini, D. Catarzi, F. Varano, S. Calenda, C. Ceni, G. Vagnoni, V. Colotta, E. Coppi, A. M. Pugliese, N. Galeotti

**Affiliations:** ^1^ Section of Pharmacology and Toxicology, Department of Neurosciences, Psychology, Drug Research and Child Health (Neurofarba) University of Florence Florence Italy; ^2^ Section of Pharmaceutical and Nutraceutical Sciences, Department of Neurosciences, Psychology, Drug Research and Child Health (Neurofarba) University of Florence Sesto Fiorentino Italy

**Keywords:** adenosine receptors, autoimmune experimental encephalomyelitis, demyelination, multiple sclerosis, oligodendrocyte progenitor cells, pain

## Abstract

Multiple sclerosis (MS) is a chronic inflammatory disease characterised by myelin and axonal loss. Lack of remyelination is primarily attributed to the failure of oligodendrocyte progenitor cells (OPCs) to differentiate into mature oligodendrocytes. The neuromodulator adenosine can influence OPC differentiation, and by selectively stimulating A_2A_ and A_2B_ receptors (A_2A_R, A_2B_R), it inhibits OPC maturation. In the efforts of developing remyelinating and neuroprotective agents, this study evaluated the ability of a novel dual A_2A_R/A_2B_R antagonist, P626, in the experimental autoimmune encephalomyelitis (EAE) mouse model and cultured OPCs. EAE mice, 14 days after MOG_35–55_ immunisation, received intranasal administration of P626 for 2 weeks, which improved motor symptoms, as evidenced by reduced clinical scores and enhanced performance on the rotarod test, and alleviated thermal and mechanical hypersensitivity without significantly affecting body weight. In spinal cord sections, P626 protected from the reduction of Luxol Fast Blue staining and increased myelin basic protein staining in immunohistochemical analysis. Patch‐clamp experiments on cultured OPCs exposed to high extracellular adenosine concentrations demonstrated that P626 prevented the A_2A_R‐ and A_2B_R‐mediated reduction in sustained *I*_*K*_ and transient *I*_*A*_ currents, both essential for cell differentiation. In conclusion, P626 showed efficacy in reducing neurological symptoms and demyelination in an MS model.

## 1 Introduction

Multiple sclerosis (MS) is a chronic inflammatory disease of the central nervous system (CNS), where immune T cell‐mediated inflammation, demyelination and neuronal damage impair motor and sensory functions [1]. Although aetiology is still unknown, several events account for disease progression. Demyelination and neurodegeneration in the CNS are primary events, and uncontrolled movements are considered the launch symptom, followed by cognitive, emotional, motor and impairments [2]. Clinically, early motor deficits, commonly attributed to demyelination, are followed by motor neuron degeneration and consequent muscle disuse [3] A hyperalgesic status is generated by the concurrence of inflammatory and neuropathic pain [4] that, if untreated, might lead to depression and anxiety [5]. Although neuroinflammation is adequately addressed by current treatments, unfortunately, the efficacy of current therapies in producing remyelination and neuroprotection is poor [6].

Lack of remyelination in MS is attributed to a failure of oligodendrocyte progenitor cells (OPCs) to differentiate into mature oligodendrocytes (OLs) [7]. Among the substances able to influence OPC differentiation is adenosine, whose effects are mediated by four metabotropic P1 receptors: A_1_, A_2A_, A_2B_, and A_3_ receptors (A_1_Rs, A_2A_Rs, A_2B_Rs, and A_3_Rs: [8]). We previously demonstrated that the selective activation of A_2A_Rs by CGS21680 inhibits OPC maturation by decreasing voltage‐dependent K^+^ currents (*I*_*k*_), necessary for their differentiation [9]. Similarly, the A_2B_R‐selective agonist BAY60‐6583 inhibits myelin basic protein (MBP) production in OPC cultures and reduces *I*_*k*_ and, differently from the A_2A_R subtype, also *I*_*A*_ [10]. Of note, the effects of BAY60‐6583 were mimicked and occluded by the adenylyl cyclase activator forskolin, demonstrating that A_2B_R‐mediated effects on cultured OPCs are mediated by an intracellular cAMP rise [10]. Recently, by using an in vitro myelination model of rat OPC‐DRG co‐cultures, a differential role of A_2B_Rs was outlined, depending on cellular localization [11]. BAY60‐6583 decreased total MBP expression in the co‐culture because of the activation of A_2B_Rs on OPCs, but increased axonal myelination through the activation of A_2B_Rs on DRG neurons.

Importantly, the sole work investigating A_2B_Rs in EAE demonstrates that A_2B_R block by the selective antagonists CVT‐6883 or MRS1754, alleviated EAE clinical symptoms and CNS immune damage and, consistently, a less severe EAE phenotype developed in A_2B_R^−/−^ mice [12]. The same authors also reported a significant increase in A_2B_R expression in peripheral lymphoid tissues of EAE mice as well as in circulating blood leukocytes of MS patients.

Results are more uncertain about the role of A_2A_Rs in demyelinating conditions. A protective effect of systemically administered A_2A_R agonists is observed in EAE mice if treatment starts on the day of immunisation [13, 14], consistent with the well‐recognised anti‐inflammatory role of A_2A_Rs in peripheral immune cells, where its stimulation decreases cell infiltration, lymphocyte differentiation and the release of pro‐inflammatory mediators [15]. Conversely, A_2A_R agonism is reported to exacerbate EAE‐induced damage if treatment starts at disease onset [16], when A_2A_R antagonists are reported to protect from EAE‐induced damage [17, 18]. Hence, it appears that peripheral A_2A_Rs are needed to dampen inflammation in the first stages of EAE, whereas their antagonism, at symptoms exordium, protects the CNS from myelin loss and neuronal damage. Importantly, A_2A_Rs are also upregulated in MS patients' peripheral blood cells and normal appearing white matter (NAWM) and, notably, A_2A_R upregulation correlates with a higher disability score [19].

On the basis of the above evidence, it appears that central A_2A_R and A_2B_R activation are deleterious in MS and its preclinical counterpart EAE but, at the same time, peripheral A_2A_R‐mediated anti‐inflammatory effects should be preserved by opportune drug delivery, that is, by intranasal administration. Interestingly, a new compound, P626, characterised by a “mixed” pharmacology, for example, able to antagonise both A_2A_Rs and A_2B_Rs, has been recently reported [20] to efficiently block A_2A_R‐ as well as A_2B_R‐mediated responses in the rat CA1 hippocampus [21]. The administration of one compound targeting two distinct receptors might present pharmacokinetic advantages and decreased risk of side effects compared to the administration of two separate drugs, that is, A_2A_R plus A_2B_R antagonist [21]. We, thus, hypothesized that intranasal administration of the dual A_2A_R/A_2B_R antagonist P626 may protect from EAE‐induced by blocking central A_2_ receptors without interfering with peripheral A_2A_R‐mediated anti‐inflammatory effect.

Our results demonstrate that the compound acts as a functional antagonist at A_2A_Rs and A_2B_Rs in oligodendroglial cell cultures and modulates EAE‐induced neurological and motor impairment, demyelination, as well as mechanical and thermal hyperalgesia, thus representing a putative pharmacological tool to protect the CNS during demyelinating pathologies.

## 2 Materials and Methods

### 2.1 Animals

The EAE model was induced on CB57BL/6 female mice (18–20 g, 6–8 weeks of age), purchased from Envigo (Varese, Italy). The animals were housed in the Ce.S.A.L. (Centro Stabulazione Animali da Laboratorio, University of Florence). Mice were housed in standard cages with eight animals per cage and maintained at 23°C ± 1°C with a 12‐h light/dark cycle, light on at 7 a.m., fed by a standard laboratory diet and tap water ad libitum and left to acclimatise for 5 days before testing. Cages were placed in the experimental room for 24 h before behavioural tests for acclimatisation, and all tests were conducted during the light phase.

The experimental protocol was approved by the Institution's Animal Care and Research Ethics Committee (University of Florence, Italy), under licence from the Italian Department of Health (336/2022‐PR). Mice were treated in accordance with the relevant European Union (Directive 2010/63/EU, the Council of September 22, 2010, on the protection of animals used for scientific purposes) and international regulations (Guide for the Care and Use of Laboratory Animals, US National Research Council, 2011). All studies involving animals are reported in accordance with the ARRIVE guidelines for experiments involving animals [22]. All effort was taken to minimise the number of animals used and their suffering. At the end of the experimentation, mice were sacrificed by cervical dislocation for spinal cord removal for in vitro analysis. The number of animals for each experiment was based on a power analysis [23], and all groups tested included 11 animals.

### 2.2 Drugs

**FIGURE 1:**
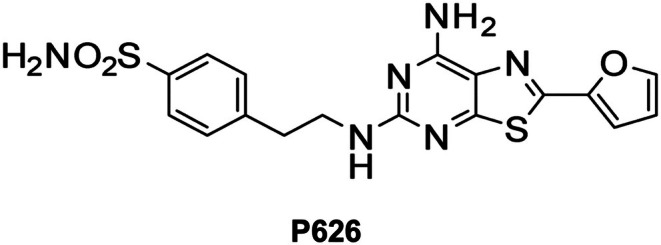
Chemical structure of P626.

The dual adenosine A_2A_AR/A_2B_R antagonist P626 (4‐(2‐((7‐Amino‐2‐(furan‐2‐yl)thiazolo[5,4‐d]pyrimidin‐5‐yl)amino)ethyl)benzenesulfonamide) (Figure 1), was synthesised as previously described (compound 2 in [20]). P626 showed high potency at both hA_2A_AR and hA_2B_AR (IC_50_ = 5.73 nM and 34 nM, respectively, cAMP assay). P626 showed a Ki = 1326 nM at hA_1_AR and a Ki = 1874 nM at hA_3_AR. All drugs were stored at −20°C as 10^3^ to 10^4^ times more concentrated stock solutions in dimethylsulfoxide (DMSO). For electrophysiological recordings, drugs were dissolved daily in the extracellular solution to the final concentration and applied by bath superfusion. For in vivo studies, P626 was solubilised in 2.5% DMSO in phosphate buffer saline (PBS) and was administered intranasally at a concentration of 10 μg/mouse. Control experiments were performed to confirm that the maximal DMSO concentration used was inactive in modulating membrane currents in electrophysiological studies (0.1%) or in producing behavioural side effects.

Adenosine; 8‐cyclopentyl‐1,3‐dipropylxanthine (DPCPX); 3‐propyl‐6‐ethyl‐5‐[(ethylthio)carbonyl]‐2‐phenyl‐4‐propyl‐3‐pyridine carboxylate (MRS1523) were purchased from Sigma‐Aldrich.

### 2.3 Intranasal Administration

The animals were lightly anaesthetised by inhalation of 2% isoflurane and placed in a supine position. Mice were administered a total of 10 μL of a solution containing 1 μg/μL of P626, with 5 μL aliquots given into each nostril [24]. Treatment was administered once daily, every other day, starting from day 14 post‐immunisation (p.i.), when symptoms were well established.

### 2.4 EAE Induction

The EAE model was induced by MOG_35‐55_ peptide (synthesised by EspiKem Srl, University of Florence, Italy), as previously described [25]. Control mice (sham) received complete Freund's adjuvant (CFA; Sigma, Milan, Italy) without antigen. The general health, body weight, locomotor coordination and noxious threshold of all mice were assessed before immunisation and every 3 days thereafter until completion of the study.

### 2.5 Behavioural Testing

**FIGURE 2:**
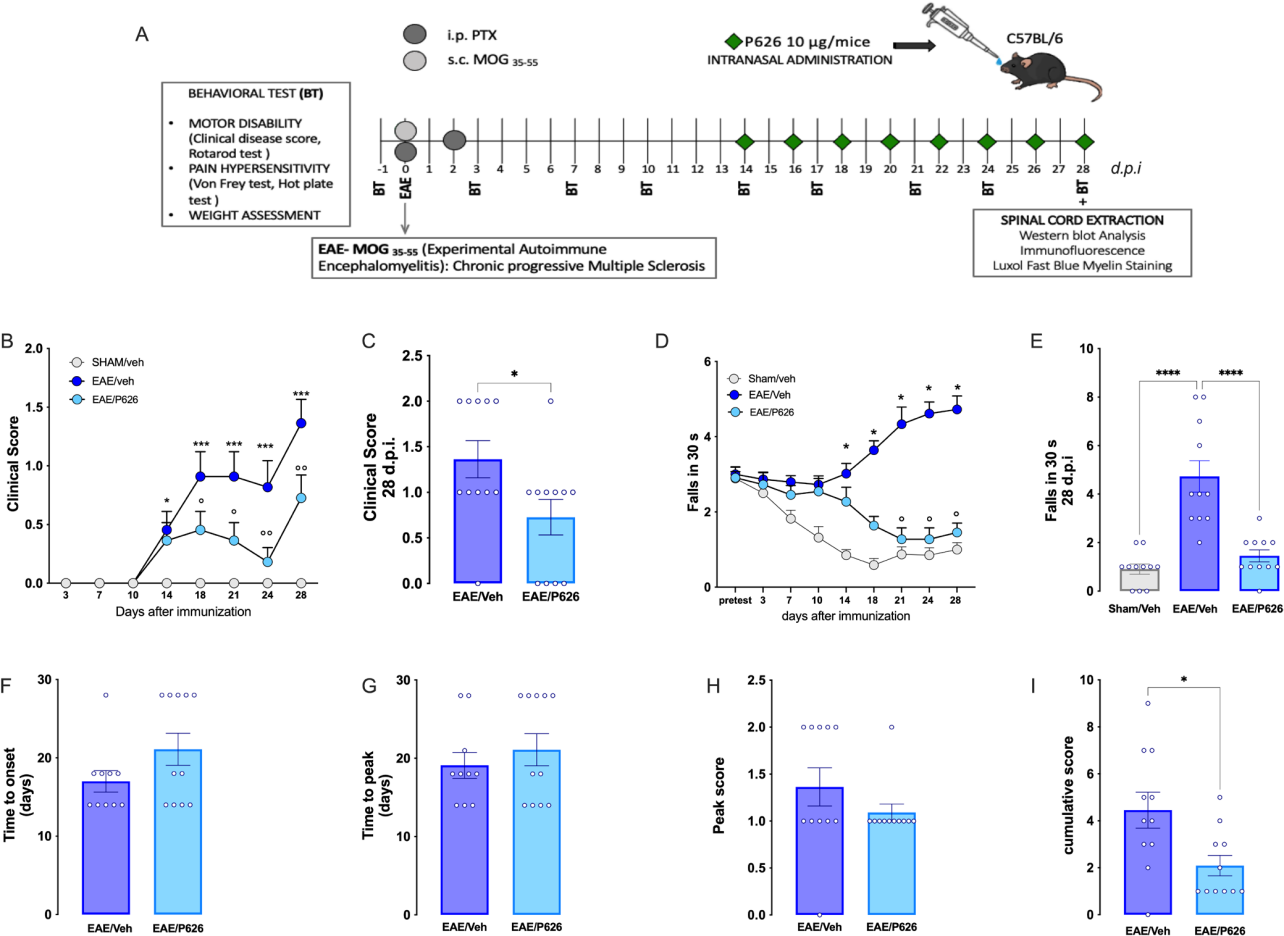
Attenuation by P626 of motor disability in EAE mice. (A) EAE immunisation protocol, treatment schedule and experimentation schedule. (B) Time course study of P626 for the clinical score values in EAE mice (two‐way ANOVA; *p < 0.05, ***p < 0.001 vs. sham/Veh; °*p* < 0.05, °°*p* < 0.01 vs. EAE/Veh) and (C) individual data points recorded 28 days post‐immunisation (d.p.i.) (unpaired Student's t‐test; *p < 0.05 vs. EAE). (D) Time course evaluation of locomotor activity on the rotarod test (two‐way ANOVA; *p < 0.05 vs. sham/Veh; °*p* < 0.05, °°*p* < 0.01 vs. EAE/Veh) and (E) and individual values on 28 d.p.i. (unpaired Student's *t*‐test; *p < 0.05 vs. EAE). Effect of P626 on time to disease onset (F), time‐to‐peak disease (G), peak disease score (H), and cumulative clinical score (I) in comparison with EAE/Veh (unpaired Student's *t*‐test; *p < 0.05 vs. EAE). Results are expressed as mean ± SEM. All tested groups included 11 animals.

In the 2 days before EAE induction, the animals were habituated to the test environment. Animals were randomly assigned to each treatment group. All tests were performed with a blind procedure, and measurements were made by the same blinded experimenter throughout the study. Behavioural testing was performed 90 min after treatment. To assess the onset and progression of pain hypersensitivity, the mice were monitored the day before immunisation for baseline values and every 3 days thereafter until the end of the experimental model (Figure 2A).

#### 2.5.1 von Frey's Test

Mechanosensory threshold was measured by using von Frey monofilaments, as described [26]. Monofilaments were applied to the plantar surface of the hind paw from a 0.008 g filament, and response was defined by paw withdrawal twice out of five completed stimuli. In case of a negative response, the next, higher‐grade filament was applied, and finally, averages of the responses were calculated.

#### 2.5.2 Hot Plate Test

Thermal threshold was measured by using the hot‐plate test, maintained at 52.5°C ± 0.1°C using a precision water bath (Ugo Basile, Varese, Italy) [27]. Endpoints included licking or shaking of the fore‐ or hind‐paws or even jumping of the animal in response to the thermal stimulus. Upon the first response, latency recording was stopped, and the animal was immediately removed from the plate. A cutoff time of 45 s was applied to prevent tissue damage.

#### 2.5.3 Rotarod Test

The mouse locomotor coordination was measured by performing the rotarod test. The Rotarod (Ugo Basile, Varese, Italy) is a device consisting of a platform and a rotating rod with a rough non‐slip surface positioned 15 cm high from the base. The rod is 30 cm long and is divided into 5 equal sections of 6 discs so that 5 animals can be evaluated at the same time. The rotation speed is 16 revolutions per min. Animals were placed on the rotating rod at the exact moment the stopwatch was operated. The integrity of the animal's motor coordination was assessed on the basis of the number of falls made from the rotating rod in a time of 30 s according to the method described by Galeotti et al. [28]. The mouse, under normal conditions, learns to move on the rod, but if it is unable to make complex movements, it falls within a few seconds.

### 2.6 Clinical Disease Score

A clinical disease score was assigned to each EAE and control mouse (sham) once daily in a blinded manner to assess the severity and extent of motor deficits. A disability scale ranging from 0 (no disability) to 5 (severe disability preceding the death of the animal) with half‐point gradations was used [25]. In any case, mice that reached a score of 3.5 were excluded from the study.

### 2.7 Western Blot Analysis

The lumbar spinal cord was removed 28 days p.i., and samples were homogenised with a pestle in a homogenization buffer. The homogenate was centrifuged at 12,000 RPM for 30 min at 4°C; the low‐speed pellet was discarded. Protein concentration in the supernatant was quantified using Bradford's method (protein assay kit, Bio‐Rad Laboratories, Milan, Italy). Supernatants (20 μg) were separated on 10% SDS‐PAGE and transferred to nitrocellulose membranes (120 min at 100 V) using standard procedures. Blots were incubated overnight at 4°C with a specific antibody against MBP (1:1000; Cell Signalling Technology, Danvers, MA, USA), NG2 (1:1000, Abcam, Cambridge, UK), CD4 (1:500, SantaCruz Biotechnology, Dallas, Texas, USA). After being washed with PBS containing 0.1% Tween, the nitrocellulose membrane was incubated with goat anti‐rabbit or anti‐mouse horseradish peroxidase‐conjugated secondary antisera (1:3000, Jackson ImmunoResearch Labs, West Grove, PA, USA) and left for 2 h at room temperature (RT; 20°C–22°C). Blots were then extensively washed and developed using an enhanced chemiluminescence detection system (ChemiDoc Imaging Systems, Bio‐Rad, Milan, Italy) and signal intensity (pixels/mm^2^) quantified (ImageJ 2.14, NIH, Bethesda, MD, USA). The exposure and development time used were standardised for all blots. For each sample, signal intensity was normalised against GADPH, and the acquired images were quantified using ImageJ 2.14 software. Measurements of control samples were assigned a relative value of 100%.

### 2.8 Immunofluorescence

Experiments were performed on the lumbar portion of the spinal cord. Samples have been fixed in formalin at 4% for 24 h, dehydrated in EtOH, included in paraffin, and finally cut into 10 μm sections. The primary antibody used was MBP (1:200; Cell Signalling Technology, Danvers, MA, USA). After rinsing in PBS containing 0.01% Triton–X–100, sections were incubated in secondary antibodies labelled with Invitrogen Alexa Fluor 488 (Jackson ImmunoResearch Labs, West Grove, PA, USA) at RT for 2 h. Sections were cover slipped using a VECTASHIELD HardSet Mounting Medium with DAPI (H‐1500, Vector Laboratories, Newark, CA, USA). A Leica DM6000B fluorescence microscope equipped with a DFC350FX digital camera with appropriate excitation and emission filters for each fluorophore was used to acquire representative images. Images were acquired with 35 to 340 mm objectives using a digital camera. The immunofluorescence intensity was calculated using Image J 2.14 on randomly selected sections per animal (20× magnification; identical acquisition settings across samples) by an experimenter blinded to the experimental groups. For each animal, 6–8 sections were averaged to yield a single value per region (five to six animals per group).

### 2.9 Luxol Fast Blue (LFB)

The LFB staining was performed on 10‐μm spinal cord sections as previously described [25]. The slides were observed on a Leica DM IL LED FLUO. Blue intensity was measured using ImageJ 2.14.

### 2.10 Cell Cultures

Purified cortical OPC cultures were prepared from Wistar rat pups (p1–2) cortices as described [10]. After mechanical and enzymatical dissociation, cells were suspended in Dulbecco's modified eagle's medium (DMEM) containing 20% fetal bovine serum (FBS), 4 mM L‐glutamine, 1 mM Na‐pyruvate, 100 U/mL penicillin, 100 U/mL streptomycin (all products are from EuroClone, Milan, Italy), and plated in poly‐L‐lysine coated T75 flasks. After 7 days, OPCs were detached by differential shaking [29] and plated onto poly‐DL‐ornithine‐coated (final concentration: 50 μg/mL, Sigma‐Aldrich) glass coverslips laid in 24 multiwell chambers (l0^4^ cells/well). OPCs were maintained in Neurobasal medium (Thermo Fisher Scientific, Milan, Italy) containing 2% B27 (ThermoFisher, Waltham, MA, USA), 4 mM L‐glutamine, 100 U/mL penicillin, 100 U/mL streptomycin, 10 ng/mL platelet‐derived growth factor‐BB and 10 ng/mL basic fibroblast growth factor (both from PeproTech, London, UK).

### 2.11 Electrophysiology

Whole‐cell patch‐clamp recordings were performed at RT on purified primary OPC cultures [10]. Cells were transferred to a recording chamber (1 mL) mounted on an inverted microscope (Olympus CKX41, Milan, Italy) and superfused at 1.5 mL/min with standard extracellular solution (mM): HEPES 5, D‐glucose 10, NaCl 140, KCl 5.4, MgCl_2_ 1.2 and CaCl_2_ 1.8 (pH 7.3 with NaOH). The selective A_1_R and A_3_R antagonists, DPCPX and MRS1523 (both 100 nM), were added to isolate A_2A_R‐ and A_2B_R‐mediated responses. Borosilicate glass electrodes (Harvard Apparatus, Massachusetts, USA) shaped by a Sutter Instruments puller (model P‐87) to a final tip resistance of 4–7 MΩ, were filled with the following intracellular solution (mM): K‐gluconate 130, NaCl 6, MgCl_2_ 2, Na_2_‐ATP 2, Na_2_‐GTP 0.3, EGTA 0.6, HEPES 10 (pH 7.4 with KOH). Data were acquired with an Axopatch 200B amplifier (Axon Instruments, CA, USA), low‐pass filtered at 10 kHz and analysed with pClamp 9.2 software (Axon Instruments, CA, USA). Drugs were applied by superfusion with a six‐way perfusion valve controller (Harvard Apparatus, Holliston, MA, USA).

Cells were voltage‐clamped at −70 mV. Series resistance, membrane resistance and membrane capacitance were routinely measured by hyperpolarising voltage pulses (from −60 to −70 mV). A voltage ramp protocol (800 ms depolarisation from −120 to +80 mV) was recorded every 15 s [10]. Depolarising voltage‐step protocols (from −40 to +80 mV, 10 mV steps), preceded by a 100 ms pre‐step potential (*V*_pre_) at −80 mV or −40 mV, were run to elicit a mixture of *I*_*A*_ and *I*_*k*_, (*V*_pre_ = −80 mV) or only the latter (*V*_pre_ = −40 mV). Net *I*_*A*_ current was then obtained in each cell by off‐line digital subtraction. In averaged results, current amplitude (pA) is expressed as current density (pA/pF) after normalisation to the respective membrane capacitance.

### 2.12 Statistical Analysis

The data and statistical analysis comply with the recommendations on experimental design and analysis in pharmacology [30]. Results are expressed as mean ± SEM. For electrophysiological analysis, Student's paired 2‐tailed *t*‐tests, or one‐way ANOVA followed by Bonferroni's post hoc analysis, were performed, as appropriate. Repeated measures of two‐way analysis of variance (ANOVA) followed by the Bonferroni test were used to compare locomotor behaviour and pain behaviours between immunised and sham mice. For behavioural assays, all tested groups comprised 11 animals. For biochemical and histological experiments, sample sizes subjected to statistical analysis had five samples per group (*n* = 5), where *n* is equal to the number of independent values. The level of significance was set to *p* < 0.05. Outliers were identified and excluded from each experimental set using the ROUT method [31]. The computer program GraphPad Prism, version 10.3 (GraphPad Software Inc., San Diego, CA), was used.

## 3 Results

### 3.1 P626 Ameliorates Motor Symptoms in EAE Mice

During the first 10 days p.i., no significant differences in EAE clinical score and locomotor activity were observed between EAE and sham groups. Fourteen days p.i. an increased clinical score was found in EAE mice that progressively worsened up to the end of the experimentation (Figure 2B,C). This corresponded to a greater number of falls from the rotarod (Figure 2D,E). Repeated intranasal administration of P626 for 2 weeks reduced the clinical score values (Figure 2B,C) and ameliorated the locomotor impairment by reducing the number of falls up to control values (Figure 2D,E). A more detailed analysis of the effect on motor symptoms showed that, although not significant, P626‐treated EAE mice showed a trend toward a prolonged time to disease onset (Figure 2F) and to reach peak clinical score (Figure 2G) than untreated EAE mice, along with a tendency to reduced maximum scores (Figure 2H). Conversely, P626‐treated mice showed a significantly lower cumulative score (Figure 2I).

### 3.2 P626 Reduces Pain Hypersensitivity in EAE Mice

**FIGURE 3:**
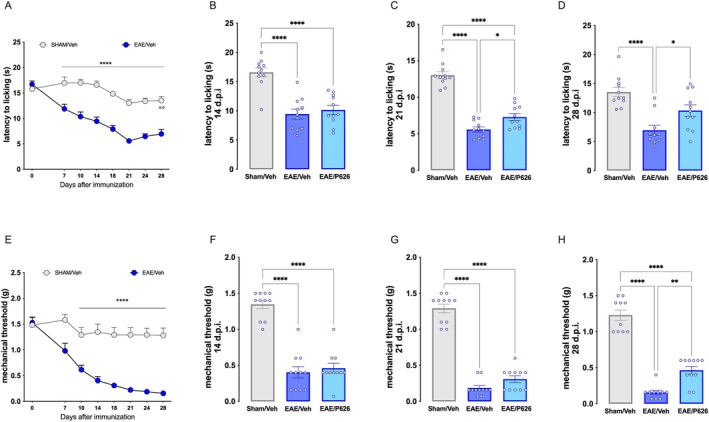
Attenuation by P626 of pain hypersensitivity in EAE mice. (A) Time course evaluation of the thermal nociceptive phenotype (hot plate test) progression in EAE mice (EAE/Veh) compared to SHAM mice (SHAM/Veh). (two‐way ANOVA; ****p < 0.0001 vs. SHAM/Veh). Effect of the three experimental groups SHAM/Veh, EAE/Veh, and EAE mice treated with compounds P626 (EAE/P626) on thermal hypersensitivity on day 14 (B), 21 (C) and 28 (D) p.i. (One‐way ANOVA; *p < 0.05, ****p < 0.0001). (E) Time course evaluation of the progression of the mechanical nociceptive phenotype (von Frey filaments) in EAE mice (EAE/Veh) compared to SHAM mice (SHAM/Veh). (two‐way ANOVA; ****p < 0.0001 vs. SHAM/Veh). Effect of P626 on mechanical hypersensitivity in EAE mice, compared to SHAM/Veh and EAE/Veh, evaluated on day 14 (F), 21 (G) and 28 (H) p.i. (One‐way ANOVA; **p < 0.01, ****p < 0.0001 vs. EAE). Results are expressed as mean ± SEM. All tested groups included 11 animals.

EAE mice developed thermal hyperalgesia (Figure 3A) and mechanical allodynia (Figure 3E) starting from day 7 post‐immunisation (p.i.), which progressively worsened, peaking at day 21 p.i. and remaining unchanged until the end of the experiment (day 28). P626‐treated EAE mice showed a progressive reduction in pain hypersensitivity from the start of the treatment (day 14 p.i.; Figure 3B,F), as indicated by pain threshold values recorded on day 21 p.i. (after 1 week of treatment; Figure 3C,G) and on day 28 p.i. (end of treatment; Figure 3D,H), when P626 significantly attenuated both thermal hyperalgesia and mechanical allodynia.

### 3.3 Lack of Effect of P626 on Body Weight

**FIGURE 4:**
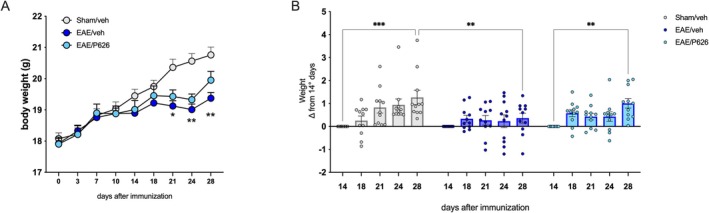
Lack of effect of P626 on body weight. (A) Evaluation of the time course of body weight of SHAM/Veh, EAE/Veh and EAE/P626 mice (two‐way ANOVA; *p < 0.05, **p < 0.01 vs. corresponding sham/veh). (B) Body weight gain values expressed as a difference from 14 d.p.i. for SHAM/Veh, EAE/Veh and EAE/P626 mice. (two‐way ANOVA; **p < 0.01, ***p < 0.001). Results are expressed as mean ± SEM. All tested groups included 11 animals.

Control mice progressively gained body weight over the 28‐day duration of the study. In contrast, EAE mice showed no variation in body weight starting from day 14 p.i., resulting in a significant difference compared to the control group by day 21 p.i. (Figure 4A). Evaluation of body weight gain as a difference from day 14 p.i. further confirmed that, although a trend toward weight gain was observed in the treatment group by day 28, P626‐treated EAE mice exhibited overall body weight values not significantly different from those of EAE mice (Figure 4A,B).

### 3.4 P626 Protects EAE Mice From Demyelination

**FIGURE 5:**
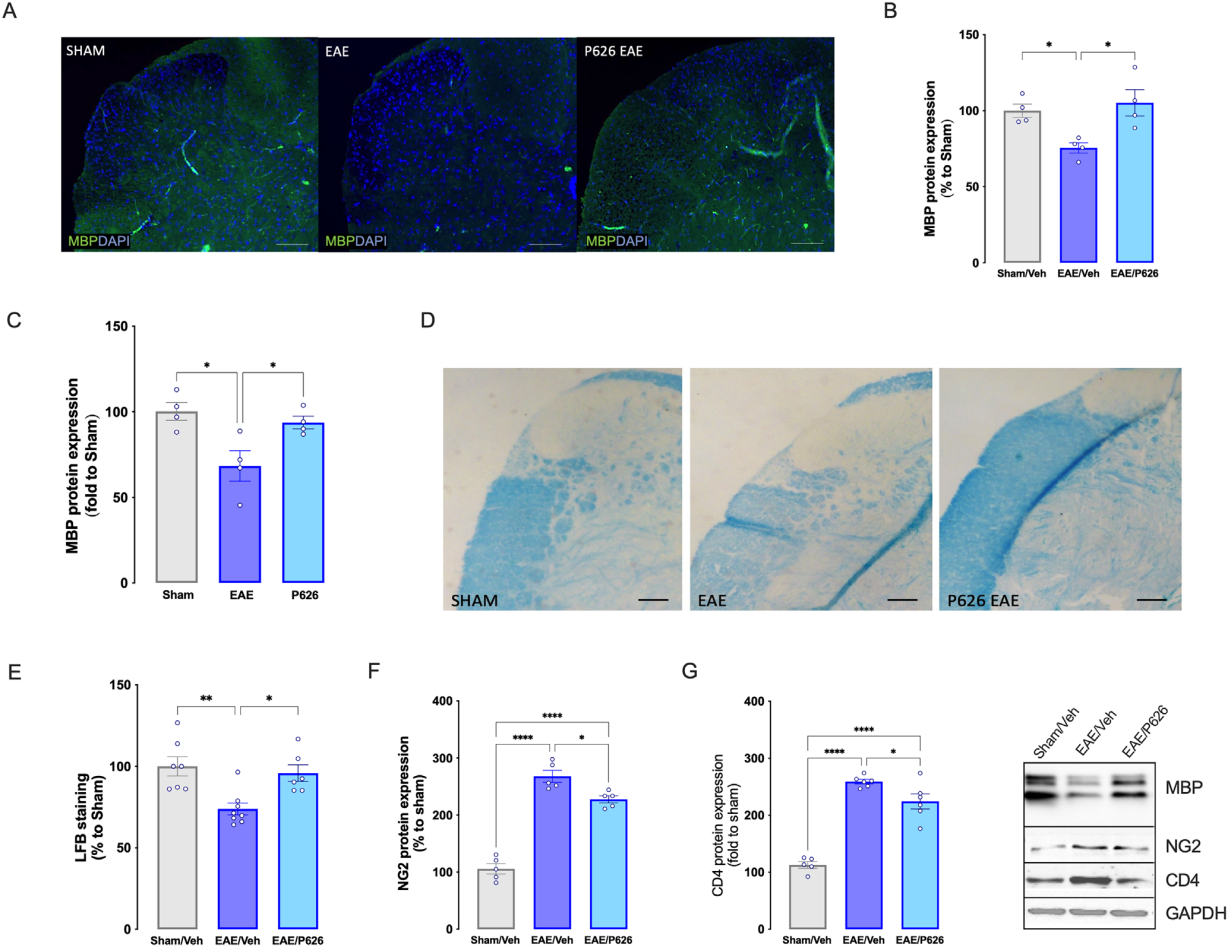
Evaluation of myelination in the lumbar spinal cord. Immunostaining representative images (A) and quantification analysis (B) of MBP protein of spinal cord tissue of SHAM/Veh, EAE/Veh, EAE/P626 (green staining corresponds to MBP expression, blue staining corresponds to DAPI expression) (One‐way ANOVA *p < 0.05). (C) MBP protein expression in EAE mice and effect of P626 treatment (One‐way ANOVA *p < 0.05). Data are the mean ± SEM of four individual experiments. (D) representative images and (E) quantitative analysis of Luxol Fast Blue (LFB) colorimetric assay on spinal cord tissue of SHAM/Veh, EAE/Veh, EAE/P626 (one‐way ANOVA *p < 0.05, **p < 0.01). Scale bar = 100 μm. EAE elevated NG2 (F) and CD4 (G) protein expression that was attenuated by P626 treatment (one‐way ANOVA *p < 0.05, ****p < 0.0001). Representative blots are reported. Results are expressed as mean ± SEM. Data are collected on 28 d.p.i.

Immunohistochemical experiments were performed to assess demyelination in the spinal cord of EAE mice. MBP staining (Figure 5A) and quantification (Figure 5B) showed a significant reduction in EAE mice 28 days after EAE induction. P626 administration significantly counteracted MBP reduction, restoring control values (Figure 5A,B). The same results were obtained by MBP quantification analysis of western blot experiments (Figure 5C). EAE mice showed elevated myelin loss following LFB staining that was reduced by P626 treatment (Figure 5D,E). Several findings indicate that OPCs fail to differentiate into mature OLs in an inflammatory microenvironment. Consequently, immature OL‐lineage cells accumulate in demyelinated lesions and remyelination remains inefficient [32]. Consistently, we observed elevated OPC levels in EAE that were attenuated by P626 (Figure 5F). Recruitment of OPCs to the demyelinated lesion temporally and spatially overlaps with the persistence of CD4+ T cells [33], suggesting that OPCs may be influenced by changes in the microenvironment caused by T cells. CD4+ T cells of the Th1 and Th17 lineage have long been recognised to play a key role in the onset and progression of MS; thus experiments to determine the content of CD4 were performed. Increased CD4 protein expression was observed in the EAE group compared with sham samples, whereas P626 treatment reduced it (Figure 5G).

### 3.5 P626 Prevents A_2A_R‐ and A_2B_R‐Mediated Adenosine Effects in Cultured OPCs

Patch‐clamp experiments were performed on cultured OPCs in order to test the functional antagonism of P626 on A_2A_R‐ and A_2B_R‐mediated effects of adenosine on voltage‐dependent K^+^ currents. Previous works characterised the effect of selective A_2A_R [34] or A_2B_R [10] activation on oligodendroglial cells, consisting of the reversible inhibition of TEA‐sensitive sustained *I*_*k*_ and, for the A_2B_R only, 4‐AP‐sensitive transient *I*_*A*_ (for a review, see Cherchi et al. [35]). In the present research, adenosine was applied at a concentration of 50 μM, sufficient to activate both the high‐affinity A_2A_R as well as the low‐affinity A_2B_R, in the continuous presence of A_1_R and A_3_R selective antagonists DPCPX and MRS1523 (100 nM both), respectively.

**FIGURE 6:**
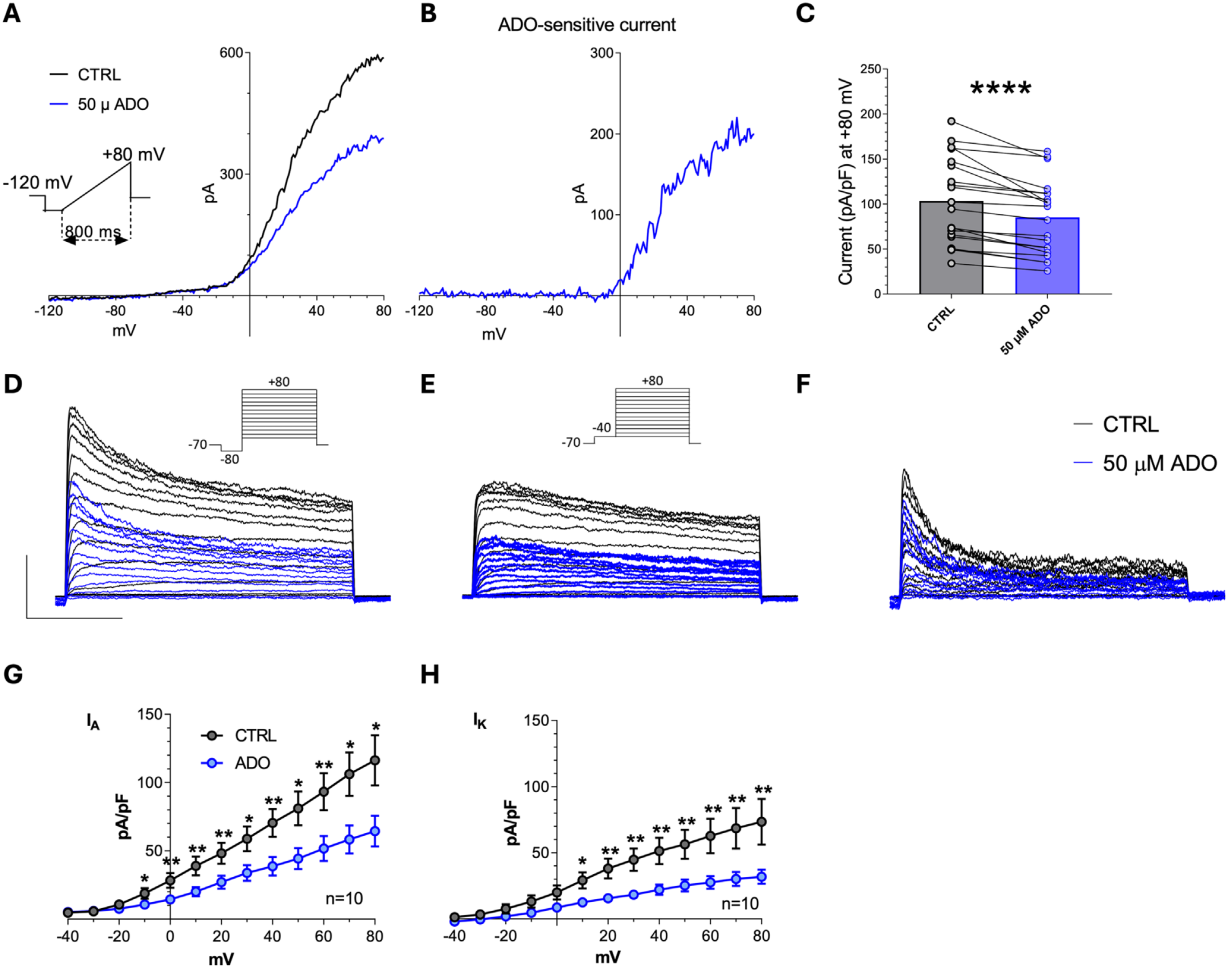
Adenosine, by activating A_2A_Rs and A_2B_Rs, inhibits K^+^ currents in purified primary OPC cultures. (A) Original patch‐clamp current traces evoked by a voltage ramp protocol (from −120 to +80 mV, 800 ms: Upper inset) in a typical OPC before (CTRL, black trace) or after 5 min application of adenosine (ADO: 50 μM; grey trace). (B) Net ADO‐sensitive current, obtained by subtracting the trace recorded in ADO from the control ramp, in the same cell. (C) Pooled data of current amplitude at +80 mV recorded in the absence (CTRL) or presence of 50 μM ADO (*n* = 20). ****p < 0.0001; Paired Student's *t*‐test. (D, E) Original current traces evoked by two different voltage‐step protocols (from −40 to +80 mV, Vpre = −80 mV; 200 ms: Inset in D; or from −40 to +80 mV, Vpre = −40 mV; 200 ms: Inset in E) in a representative OPC before (CTRL: Black traces) or after 5 min application of ADO (50 μM, grey traces). Calibration bars: 100 ms; 500 pA. (F) Net *I*_*A*_ current in the same OPC obtained by subtraction of traces reported in D and F. (G, H) Averaged current‐to‐voltage relationship (I–V plot) of peak, transient, *I*_*A*_ currents (G) or steady‐state, sustained, *I*_*k*_ currents (H) recorded in the absence (CTRL: Black circles) or presence (grey circles) of 50 μM ADO in 10 cells investigated. *p < 0.05; **p < 0.01; Paired Student's *t*‐test. All experiments were performed in the presence of the A_1_R and A_3_R antagonists, DPCPX and MRS1523, respectively (all 100 nM). Results are expressed as mean ± SEM.

**FIGURE 7:**
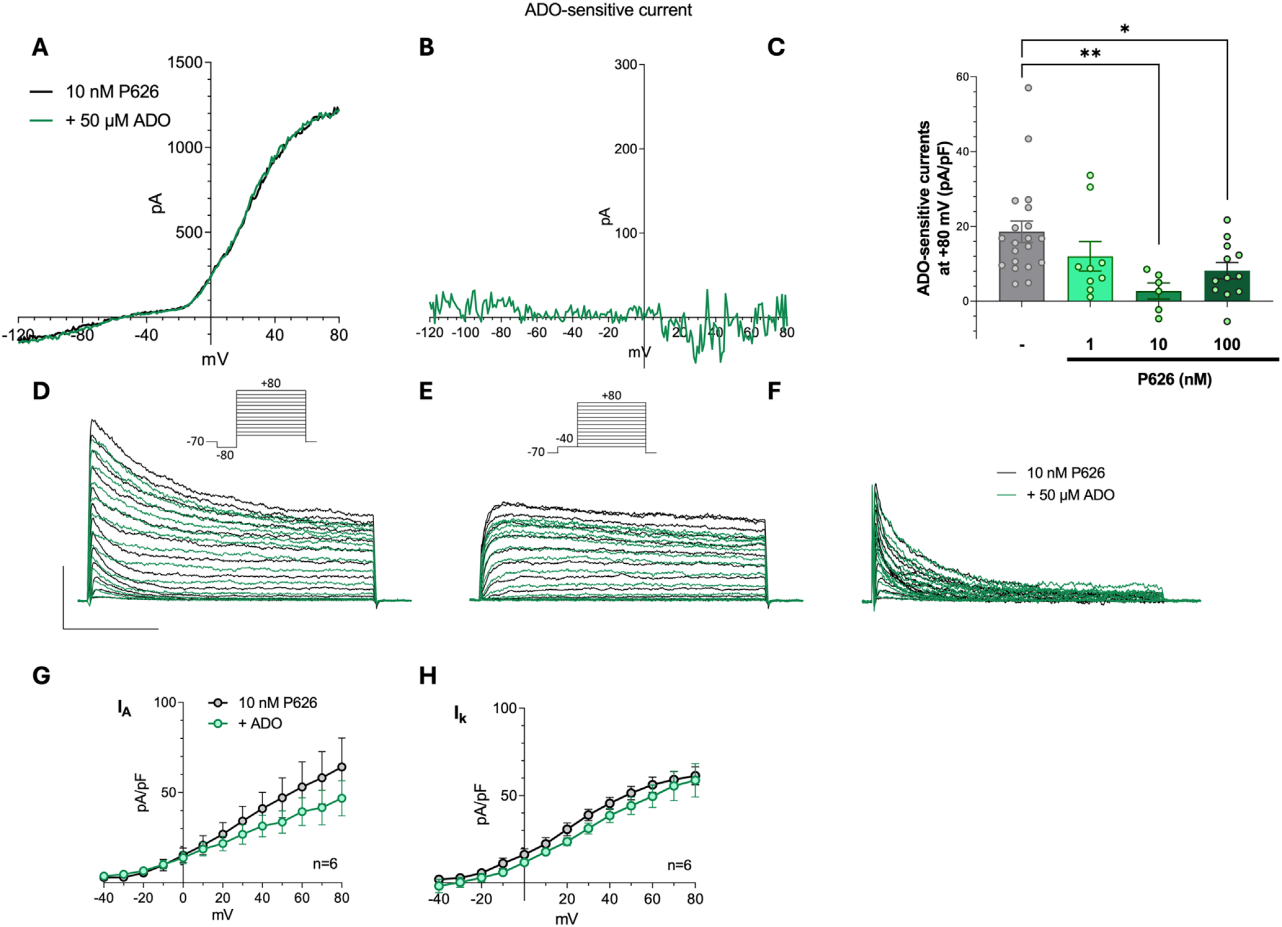
The dual A_2A_R/A_2B_R antagonist, P626, counteracts adenosine‐mediated reduction of K^+^ currents in cultured OPCs. (A) Original patch‐clamp current traces evoked by a voltage ramp protocol (from −120 to +80 mV, 800 ms) in a typical OPC before (black trace) or after 5 min application of 50 μM adenosine (ADO: Green trace) in the presence of 10 nM P626. (B) Net ADO‐sensitive current, obtained by subtracting the trace recorded in ADO+P626 from that recorded in the presence of P626 alone, in the same cell. (C) Pooled data of ADO‐sensitive currents at +80 mV in the absence (−) or presence of different concentrations of P626. *p < 0.05; **p < 0.01; one‐way ANOVA, Bonferroni post‐test. (D, E) Original current traces evoked by two different voltage‐step protocols (see insets) in a representative OPC before (black traces) or after 5 min application of ADO (50 μM, green traces). Calibration bars: 100 ms; 500 pA. (F) Net *I*_*A*_ current in the same OPC obtained by subtraction of traces reported in D and E. (G, H) Averaged current‐to‐voltage relationship (I–V plot) of peak, transient, *I*_*A*_ currents (G) or steady‐state, sustained, *I*_*k*_ currents (H) recorded in the absence (black circles) or presence (grey circles) of 50 μM ADO in 10 cells investigated. *p < 0.05; **p < 0.01; Paired Student's *t*‐test. All experiments were performed in the presence of the A_1_R and A_3_R antagonists, DPCPX and MRS1523, respectively (all 100 nM). Results are expressed as mean ± SEM.

As shown in Figure 6A, adenosine (ADO; 50 μM, 5 min application) decreased voltage‐dependent overall currents evoked by a depolarising voltage ramp protocol (−120 to +80 mV, 800 ms: inset in Figure 1A) in purified primary OPC cultures. As we previously demonstrated, the vast majority of currents evoked by this ramp protocol are K^+^ currents as they are abrogated by Cs^+^ replacement [10]. Adenosine‐sensitive current was obtained in each cell by subtraction of the ramp current recorded in adenosine from the control ramp (Figure 6B). The effect of 50 μM adenosine, in conditions of A_1_R and A_3_R block, was statistically significant in 20 cells tested (Figure 6C: from 103.3 ± 10.7 in control to 85.1 ± 9.3 pA/pF in adenosine, ****p < 0.0001, paired Student's *t*‐test). When opportune voltage step protocols were applied (see methods and upper panels in Figure 6D,E), we found that 50 μM adenosine inhibited both sustained *I*_*k*_ and transient *I*_*A*_ currents (Figure 6D–H), in line with combined activation of A_2A_Rs and A_2B_Rs, the latter being the only one able to inhibit *I*_*A*_ in cultured OPCs [10]. When applied in the presence of the dual A_2A_R/A_2B_R antagonist P626, adenosine‐mediated reduction of ramp‐evoked currents (Figure 7A–C), as well as of step‐evoked *I*_*k*_ and *I*_*A*_ currents (Figure 7D–H), was prevented.

## 4 Discussion

Experiments were conducted using the mouse model of EAE, which recapitulates, albeit with some limitations, key events observed in MS patients: neuroinflammation, demyelination and neurodegeneration [36]. Inflammation and demyelination are evident in both the CNS and peripheral systems. Mechanical allodynia develops around 21 days p.i., which is attributed to lymphocyte infiltration in the dorsal root ganglia (DRG). A concomitant decrease in MBP content in the sciatic nerve is observed in EAE mice as early as day 14 p.i. [37]. These events are followed by altered DRG neuron excitability at day 21 p.i. [38], and at day 28 p.i., a range of behavioural and cognitive impairments, including anhedonia, anxiety, and stress, are revealed through behavioural tests. In the brain, impaired hippocampal synaptic plasticity (long‐term potentiation: LTP) is detected between days 15–18 p.i. [39] and is attributed to peripheral T cell infiltration and interleukin‐17A (IL‐17A) release in this brain area [40, 41]. Repeated intranasal administration of P626 for 2 weeks attenuated the main symptoms of EAE. Motor disability was progressively reduced, as indicated by lower clinical score values, and motor coordination (rotarod performance) improved at the end of the 28‐day period of observation. These results are consistent with previous findings demonstrating the ability of A_2A_R antagonists to protect against motor symptoms in EAE [17, 42, 43]. Furthermore, our findings support the hypothesis that A_2B_R blockade may play a protective role, as evidenced by the efficacy of an A_2B_R antagonist in reducing EAE‐related motor disability [12].

Among the many sensory disturbances experienced by MS patients, pain is particularly disabling, with more than 90% of MS patients reporting bodily pain. Furthermore, 85% of these patients report that the pain is severe enough to cause functional disability [44]. Additionally, a very high percentage of MS patients suffer from neuropathic pain [45], which is one of the most difficult‐to‐treat pain conditions and negatively impacts general health, energy, mental health, and social functioning, significantly affecting daily life.

Repeated treatment with P626 in EAE mice attenuated both mechanical allodynia and thermal hyperalgesia on day 28 p.i. To the best of our knowledge, this is the first evidence of a dual A_2A_R/A_2B_R antagonist providing protective activity against both motor and pain‐related symptoms. A clear limitation of these approaches is the confounding effect of motor disability, which is characteristic of clinical EAE and interferes with the assessment of pain behaviours because of the absence of withdrawal reflexes during paralysis. In our experimental EAE protocol, motor disability was not severe enough to completely interfere with hindpaw movement, which might have made it difficult to detect any changes in pain thresholds. Indeed, the motor deficits observed at severe clinical scores (3 or higher) may render the assessment of mechanical allodynia or thermal hyperalgesia unreliable [46, 47, 48].

Patients suffering from neuropathic pain tend to have more severe MS than those without pain [49], suggesting a potential correlation between neuropathic pain and motor disability. We further speculate that P626 may prevent the altered excitability of DRG nociceptors, as A_2B_R activation has been shown to increase action potential firing in these cells [11].

Recent evidence has linked neuropathic pain in MS to demyelination [50]. Therefore, we investigated the ability of P626 to attenuate myelin loss. Analysis of spinal cord samples from EAE mice showed significant demyelination, as evidenced by reduced Luxol Fast Blue staining and MBP expression. P626 treatment counteracted myelin loss, restoring basal myelin levels. A_2A_R and A_2B_R are expressed both centrally and peripherally, playing a prominent role in exacerbating neuronal and glial damage when activated in the CNS; conversely, A_2A_R attenuates the inflammatory response when activated in peripheral blood cells [51]. Previous studies reported a moderate effect of A_2A_R antagonists on peripheral proliferation and inflammation in EAE mice, compared to their stronger anti‐neuroinflammatory activity through modulation of microglial and astroglial activation [52]. Worth noticing, A_2A_R antagonists proved effective in reducing EAE symptoms only when administered at later stages of the disease. In contrast, negative effects were observed if administered during the first week post‐immunisation, when proinflammation prevails. This time‐dependent differential activity has been proposed to underlie a protective effect on demyelination and neurodegeneration by A_2A_R blockade independently of its action on peripheral blood cells [53]. To investigate the protective effects of P626 on demyelination, it was essential to focus on central processes, excluding potential confounding effects from peripheral mechanisms. For this reason, the dual antagonist was administered intranasally, a non‐invasive delivery route that facilitates brain penetration.

Lineage‐tracing experiments have demonstrated that OPCs are able to differentiate into new OLs after demyelination (e.g., [54]). However, OPCs fail to differentiate into mature OLs in an inflammatory microenvironment (e.g., elevated levels of cytokines and activated immune cells as occurs in EAE). Consequently, immature OL‐lineage cells accumulate in demyelinated lesions and remyelination remains inefficient [32]. Consistently, we observed elevated OPC levels in EAE that were attenuated by P626. These findings further suggest that P626 might attenuate demyelination by acting on OPCs. To deepen our understanding of the mechanisms underlying the beneficial effects of P626 during demyelination, we tested the compound on purified primary OPC cultures in conditions of high extracellular adenosine, as found in MS patients and EAE mice [55, 56, 57]. Adenosine is known to inhibit K^+^ currents through the activation of Gs‐coupled receptors. In particular, selective A_2A_R activation with CGS21680 inhibits sustained, TEA‐sensitive *I*_*K*_ [9], whereas the A_2B_R agonist BAY60‐6583 inhibits both *I*_*K*_ and transient, 4‐AP‐sensitive, *I*_*A*_ currents [10]. Of note, *I*_*K*_ inhibition causes reduced OPC differentiation and MBP expression [34, 58] and, indeed, either CGS21680 or BAY60‐6583 inhibit OPC maturation [9, 10]. In this study, we applied 50 μM adenosine in the presence of selective A_1_R and A_3_R blockers to isolate A_2A_R‐ and A_2B_R‐mediated responses in OPCs. Adenosine inhibited both sustained *I*_*K*_ and transient *I*_*A*_ currents (Figure 6D–H), an effect consistent with A_2A_R and A_2B_R activation, as confirmed by a significant block in the presence of the dual A_2A_R/A_2B_R antagonist, P626.

Beyond a direct action of P626 on oligodendroglial cells, the compound could also exert neuroprotective effects in EAE mice, also by targeting A_2A_R and/or A_2B_R expressed on neurons. Indeed, it is known that both adenosine receptors are expressed at presynaptic terminals, where they promote glutamate release [59, 60] and exacerbate ischemic‐like neuronal damage induced by oxygen and glucose deprivation (OGD) in acute hippocampal slices [61, 62]. Accordingly, we recently demonstrated that P626 prevents A_2A_R‐ as well as A_2B_R‐mediated increase in presynaptic glutamate release induced by each respective agonist [21] and protects CA1 neurons from OGD‐induced damage [21]. Hence, beyond its protective effects on OPCs, A_2A_R‐ as well as A_2B_R blockade on neurons could be beneficial in conditions of high extracellular adenosine, such as those achieved during EAE [55, 57], by counteracting excitotoxic glutamate overload.

Present data demonstrate that the dual antagonist P626 efficiently prevented A_2A_R‐ and A_2B_R‐dependent responses in OPCs, a mechanism consistent with its protective effects in EAE mice. Thus, we propose that P626, administered intranasally in EAE mice, may counteract the anti‐differentiating effects induced by adenosine through A_2A_Rs and A_2B_Rs activation in OPCs, thereby promoting their differentiation. Even though further studies are needed to directly link P626 with remyelination, these findings, along with the data presented above, show that P626 treatment mitigated myelin loss and restored basal myelin levels in spinal cord samples from EAE mice.

In conclusion, P626 shows potential as a compound for attenuating neurological symptoms and demyelination in MS patients, warranting further investigation to confirm its therapeutic efficacy.

## Author Contributions

**M. Morozzi:** data curation (equal), formal analysis (lead), investigation (lead), methodology (equal). **F. Cherchi:** data curation (equal), formal analysis (equal), investigation (equal), methodology (equal). **C. Sasia:** formal analysis (equal), investigation (equal), methodology (equal). **L. Frulloni:** formal analysis (equal), investigation (equal), methodology (equal). **G. Videtta:** formal analysis (equal), investigation (equal), methodology (equal). **M. Venturini:** formal analysis (equal), investigation (equal), methodology (equal). **D. Catarzi:** funding acquisition (equal), writing – review and editing (equal). **F. Varano:** resources (equal), writing – review and editing (equal). **S. Calenda:** investigation (equal), methodology (equal). **C. Ceni:** formal analysis (equal), methodology (equal). **G. Vagnoni:** investigation (equal), methodology (equal). **V. Colotta:** conceptualization (equal), funding acquisition (equal), writing – review and editing (equal). **E. Coppi:** investigation (equal), methodology (equal), writing – review and editing (equal). **A. M. Pugliese:** conceptualization (equal), funding acquisition (equal), writing – review and editing (equal). **N. Galeotti:** conceptualization (equal), funding acquisition (equal), methodology (lead), project administration (lead), resources (equal), supervision (lead), validation (lead), visualization (lead), writing – original draft (lead), writing – review and editing (equal).

## Conflicts of Interest

The authors declare no conflicts of interest.

## Data Availability

The data presented in this study are available on request from the corresponding author.
